# Transinfection of buffalo flies (*Haematobia irritans exigua*) with *Wolbachia* and effect on host biology

**DOI:** 10.1186/s13071-020-04161-8

**Published:** 2020-06-10

**Authors:** Mukund Madhav, Geoff Brown, Jess A. T. Morgan, Sassan Asgari, Elizabeth A. McGraw, Peter James

**Affiliations:** 1 Queensland Alliance for Agriculture and Food Innovation (QAAFI), The University of Queensland, Brisbane, QLD 4072 Australia; 2 Department of Agriculture and Fisheries, Brisbane, 4001 Australia; 3 Australian Infectious Disease Research Centre, School of Biological Sciences, The University of Queensland, Brisbane, QLD 4072 Australia; 4 Department of Entomology, Center for Infectious Disease Dynamics, Pennsylvania State University, University Park, PA 16802 USA

**Keywords:** *Wolbachia*, *Haematobia*, Biocontrol, Veterinary ectoparasite, Endosymbiont, Pest management

## Abstract

**Background:**

Buffalo flies (*Haematobia irritans exigua*) (BF) and closely related horn flies (*Haematobia irritans irritans*) (HF) are invasive haematophagous parasites with significant economic and welfare impacts on cattle production. *Wolbachia* are intracellular bacteria found widely in insects and currently of much interest for use in novel strategies for the area wide control of insect pests and insect-vectored diseases. In this paper, we report the transinfection of BF towards the development of area-wide controls.

**Methods:**

Three stages of BF; embryos, pupae and adult female flies, were injected with different *Wolbachia* strains (*w*AlbB, *w*Mel and *w*MelPop). The success of transinfection and infection dynamics was compared by real-time PCR and FISH and fitness effects were assessed in transinfected flies.

**Results:**

BF eggs were not easily injected because of their tough outer chorion and embryos were frequently damaged with less than 1% hatch rate of microinjected eggs. No *Wolbachia* infection was recorded in flies successfully reared from injected eggs. Adult and pupal injection resulted in higher survival rates and somatic and germinal tissue infections, with transmission to the succeeding generations on some occasions. Investigations of infection dynamics in flies from injected pupae confirmed that *Wolbachia* were actively multiplying in somatic tissues. Ovarian infections were confirmed with *w*Mel and *w*MelPop in a number of instances, though not with *w*AlbB. Measurement of fitness traits indicated reduced longevity, decreased and delayed adult emergence, and reduced fecundity in *Wolbachia*-infected flies compared to mock-injected flies. Effects varied with the *Wolbachia* strain injected with most marked changes seen in the *w*MelPop-injected flies and least severe effects seen with *w*AlbB.

**Conclusions:**

Adult and pupal injection were the most suitable methods for transinfecting BF and all three strains of *Wolbachia* successfully replicated in somatic tissues. The *Wolbachia-*induced fitness effects seen in transinfected BF suggest potential for use of the *w*Mel or *w*MelPop strains in *Wolbachia*-based biocontrol programmes for BF.
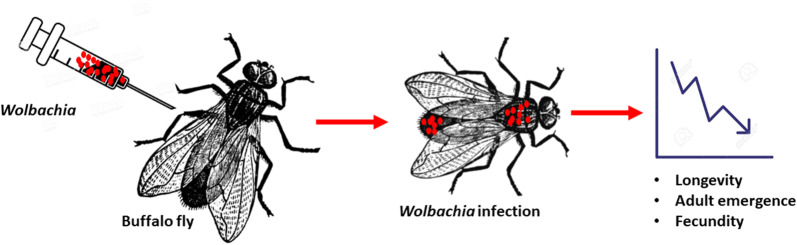

## Background

Buffalo flies (BF), *Haematobia irritans exigua* are obligate hematophagous ectoparasites of cattle [1]. They are present in the Australasian, Oriental and Palaearctic regions of the world [2]. They are very closely related to horn flies (*Haematobia irritans irritans*) (HF), which are major economic pests in North America and, more recently, South America and are also distributed widely across Europe to North Asia. Both female and male BF and HF feed 20–40 times a day on cattle and the females only leave cattle to oviposit in freshly deposited cattle manure [3]. Their blood-feeding habits result in significant economic losses by reducing milk and meat production and causing defects in cattle leather [4, 5]. Further, BF and HF infestations are a significant welfare issue with biting by the flies causing severe irritation and, in association with filarial nematodes transmitted by these flies (*Stephanofilaria stilesi* by HF and an undescribed species of *Stephanofilaria* by BF), the development of lesions that range from dry, hyperkeratotic and alopecic areas to open ulcerated sores. BF are tropical and subtropical in their distribution and are mainly pests of cattle in the northern parts of Australia [6]. However, aided by a warming climate and reduced efficiency of control because of the development of chemical resistance, they have been steadily expanding their range southward [2, 6–8].

*Wolbachia* are maternally inherited endosymbionts of insects, that are of much interest for use in the biological control of pests, most particularly as a basis for area-wide integrated control strategies for a range of insect species [9–11]. *Wolbachia* has been used in insect control programmes in two main ways. First, it has been used as a means to achieve population replacement, where *Wolbachia*-infected insects impart unique characteristics such as pathogen blocking or fitness deficits [12–15] and secondly, by the incompatible insect technique (IIT) in which *Wolbachia*-infected males released into the population cause the production of non-viable eggs, similar to the sterile male technique [10, 16–19]. The spread of a new strain of *Wolbachia* through a pest population is required for approaches that rely on population replacement, but is not desirable when *Wolbachia*-induced cytoplasmic incompatibility is used for IIT [20]. Some of the novel fitness costs induced by *Wolbachia* include decreased fecundity and male competitiveness, seen in *Anopheles stephensi* infected with *w*AlbB, life span reduction, egg mortality, delayed larval development and altered feeding behaviour seen in *Aedes aegypti* infected with *w*MelPop [21–26].

The first successful field trial of the *Wolbachia-*based IIT technique was in Myanmar in early 1960’s to eliminate a native population of *Culex quinquefasciatus* mosquitoes responsible for transmitting filariasis [27]. Following the trial success, this strategy has been widely studied in mosquito species including *Aedes polynesiensis*, *Aedes albopictus*, *Culex pipiens pallens* and in tsetse flies (*Glossina morsitans*) [10, 28–31].

Presently, both the sexes of *w*Mel-infected *Ae. aegypti* mosquitoes are being released in Australia [32], Indonesia [33] and Brazil [15] and *w*AlbB-infected *Ae. aegypti* is being released in Malaysia [34] with the aim of population replacement for the control mosquito transmitted arboviruses. Other male only releases aiming at population control by IIT include *w*Pip-infected *Ae. albopictus* in China [35] and the USA [16] and *w*AlbB-infected *Ae. aegypti* in the USA [17].

The first step towards developing *Wolbachia-*based control programmes is the establishment of *Wolbachia* transinfected lines of the target pest. The most common method used to transinfect new hosts with *Wolbachia* has been embryonic microinjection, although injection into other stages, such as adults and pupae have also given some success [36]. Of the available transinfection procedures, embryonic microinjection is mostly preferred as *Wolbachia* are directly introduced to the pole cells of pre-blastoderm embryos using a fine needle inserted at the posterior end of the egg, desirably resulting in germline and somatic cell infection. In contrast, adult injection is usually carried out into the thoracic or abdominal regions of adults where *Wolbachia* must successfully evade or overcome a number of membrane barriers and the host immune response to become established in the germinal tissues for next-generation transmission [36]. Some instances of successful use of adult microinjection to transinfect new insect strains include the transfer of *w*Mel strain to uninfected *Drosophila melanogaster*, *w*AlbA and *w*AlbB to *Ae. aegypti* and *w*Ri, *w*Mel, *w*Ha and *w*No to the leafhopper *Laodelphax striatellus* [36–39].

Buffalo flies collected from twelve locations in Australia and Indonesia were negative for *Wolbachia* infection, and this has been confirmed by more recent testing in our laboratory (unpublished data) [40]. However, *Wolbachia* appears to be ubiquitous in closely related horn flies (*H. i. irritans*) (HF) suggesting that BF will also be a competent host for *Wolbachia* [40–46]. In previous studies, *Wolbachia* has been mostly sourced from the egg of the infected species for microinjection purposes [36]. Nevertheless, using cell lines of the intended host artificially infected with *Wolbachia* as the donor source has been suggested as advantageous for obtaining a high density and host context adapted *Wolbachia* [47]. Hence, we established the HIE-18 cell line from HF to adapt *w*AlbB obtained from mosquito, *w*Mel and *w*MelPop from *Drosophila* into the *Haematobia* spp. context prior to commencing BF microinjection [48].

Here, we report the results of studies towards the establishment of lines of BF sustainably infected with the *w*AlbB, *w*Mel and *w*MelPop strains of *Wolbachia* and the dynamics and kinetics of infection in microinjected flies. The results of preliminary investigations into the related physiological costs of *Wolbachia* infection on the newly infected host BF, which are critical to considerations of the potential for use in biological control programmes, are also described.

## Methods

### Establishment of *Wolbachia*-infected cell cultures

A non-infected *Drosophila* cell line (JW18) was infected with the *w*AlbB (JW18-*w*AlbB), *w*Mel (JW18-*w*Mel) and *w*MelPop (JW18-*w*MelPop) strains of *Wolbachia* following the protocol of Herbert & McGraw [49] to first adapt them in a closely related species. JW18 cell lines infected with the three strains of *Wolbachia* were cultured in a 75 cm^2^ flask in 12 ml of Schneider’s medium supplemented with 10% FBS at 28 °C (Sigma-Aldrich, NSW, Australia). Infected JW-18 cell lines were used for isolation of *Wolbachia* for the first three adult injection and embryonic microinjection batches. The *Haematobia* embryonic cell line (HIE-18) maintained in our laboratory without the use of antibiotics were transinfected with *w*AlbB (*w*AlbB-HIE-18), *w*Mel (*w*Mel-HIE-18) and *w*MelPop (*w*MelPop-HIE-18) as above [48]. The infected HIE-18 lines were cultured in 75 cm^2^ flasks containing 12 ml of Schneider’s medium supplemented with 10% FBS at 28 °C and subcultured every 5–6 days by splitting at a ratio of 1:2 into new flasks (Sigma Aldrich, NSW, Australia). Infected HIE-18 cell lines were used as donor for all of the later embryonic, adult and pupal BF microinjections.

### *Wolbachia* isolation

*Wolbachia* were isolated from the cell lines, according to Herbert & McGraw [49]. Briefly, *w*AlbB-, *w*Mel- and *w*MelPop-infected cell lines were grown in 75 cm^2^ cell culture flasks for seven days using previously noted methods. Cells were pelleted on the eighth day by spinning at 2000×*g* and washed three times with SPG buffer (218 mM sucrose, 3.8 mM KH_2_PO_4_, 7.2 mM MK_2_HPO_4_, 4.9 mM L-glutamate, pH 7.5), sonicated on ice for two bursts of 10 s and cellular debris was removed by spinning at 1000×*g* for 10 min at 4 °C. The supernatant was passed through 50 μm and 2.7 μm acrodisc syringe filters (Eppendorf, Sydney, NSW, Australia) and centrifuged at 12,000×*g* to pellet *Wolbachia*. Finally, the pellet was suspended in 100 µl SPG buffer and used for microinjection.

### Embryonic microinjection

The different stages of BF (eggs, pupae and adults) used for microinjection were sourced from a BF laboratory colony established from field collected flies from southeast Queensland in 2012 and which had been maintained since this time at the EcoScience Precinct, Brisbane, following protocol described by James [50]. To obtain eggs of similar age for microinjection, 7–10 day-old BF were held on moist filter paper in temporary cages for 20–30 min. Newly laid eggs (40–60 min-old) were arranged on double-sided sticky tape using a paintbrush and microinjected at the posterior pole of each egg with *w*AlbB (2 × 10^8^ bacteria/ml) using a FemtoJet microinjector system (Eppendorf, Sydney, NSW, Australia). The microinjected eggs were then placed on tissue paper on the surface of manure pats to hatch. After eclosion, larvae migrated into the moist manure where they fed until pupation. Pupae were separated from the manure by flotation in water on day 7 post-injection and incubated at room temperature. Flies that emerged from the puparium by day 10 were collected and separated by sex. Females that emerged from microinjected eggs were held singly with two uninfected males for mating in small cages made of transparent acrylic pipe (6 cm diameter × 15 cm height) closed with fly mesh and a membrane feeder at the top supplying cattle blood maintained at 26 °C. A 55 cm^2^ Petri dish containing moist filter paper was placed at the base of the cages for collection of eggs deposited by the flies. Females were allowed to oviposit and the eggs were collected until the death of the flies. Freshly deceased flies were collected and tested for the presence of *Wolbachia* using real-time PCR.

### Adult microinjection

Approximately 100–150 pupae from the BF colony at the EcoScience Precinct, Brisbane, Australia, were held separately from the main colony for collection of freshly emerged female flies (2–3 h-old) for injection. The female flies were collected within 3–4 h of eclosion from the pupae, anaesthetised using CO_2_ for 30–40 s and then 2 µl of *Wolbachia* suspension (3 × 10^9^ bacteria/ml) was injected into the metathorax of each fly using a handheld micro-manipulator (Burkard Scientific, London, UK) with hypodermic needles (0.24 × 33 mm). The microinjected flies (G_0_) were blood-fed and mated with male flies at the ratio of 1:1 in small cages as described above. On day three after injection, an artificial 100 g manure pat was placed onto sand at the base of each cage. Manure pats were removed every second day and the collected eggs were reared to adults following our standard laboratory protocols. Newly hatched G_1_ female flies were mated to potentially infected males, allowed to oviposit until death and the freshly deceased G_1_ flies then tested by real-time PCR for the presence of *Wolbachia.* Depending on the results of testing, the cycle was repeated.

### Pupal microinjection

Approximately 3000–4000 eggs from colony-reared BF were incubated and the larva grown on manure to collect freshly pupated BF for microinjection (1–2 h-old). Pupae were aligned on double-sided sticky tape and injected in the third last segment at the posterior end close to germinal tissue using a FemtoJet microinjector system (Eppendorf). The microinjected pupae were then placed on moist Whatman filter paper and incubated at 27 °C until flies emerged. Freshly emerged flies were separated and placed in a cage with a maximum of five females and five males each. Eggs collected from each cage every day were tested for *Wolbachia* infection. Once infection was detected, female flies were separated into a separate single cage and eggs were collected for the G_1_ line until the flies died. Later, freshly deceased females were tested for the presence of *Wolbachia* using real-time PCR.

### *Wolbachia* diagnostic assay

A Chelex extraction protocol modified from Echeverria-Fonseca et al. [51] was used for extraction of DNA from the embryonic and adult microinjected samples. Briefly, flies were homogenised using a Mini-Beadbeater (Biospec Products, Bartlesville, Oklahoma, USA) for 5 min in 2 ml screw-cap vials with 2 g of glass beads (2 mm) and 200 µl of buffer containing 1× TE buffer and 10% Chelex®-100 (Bio-Rad Laboratories, CA, USA). Samples were then incubated overnight at 56 °C with 10 µl of Proteinase K (20 mg/ml) and dry boiled the next day for 8 min at 99.9 °C. Finally, samples were spun at 13,000×*g* for 15 min and the supernatant was stored at − 20 °C until tested. For pupal-injected samples and eggs, DNA was extracted using an Isolate II Genomic DNA extraction kit (Bioline, Sydney, NSW, Australia). DNA was amplified with strain-specific primers using a Rotor-Gene Q machine (Qiagen, Melbourn, VIC, Australia) (Table 1). Reactions were run in a total of 10 μl having 5 μl PrimeTime® Gene Expression Master Mix (IDT, VIC, Australia), 0.5 μl each of 10 µM forward and reverse primer, 0.25 μl of 5 µM probe and 3 µl of genomic DNA. *Wolbachia* DNA and DNA from uninfected adult BF were run with every batch of the samples as positive and negative PCR controls, respectively. Optimised amplification conditions for *w*Mel and *w*MelPop were 3 min at 95 °C followed by 45 cycles of 10 s at 95 °C, 15 s at 51 °C and 15 s at 68 °C. For *w*AlbB, the optimized amplification conditions were 3 min at 95 °C followed by 45 cycles of 20 s at 94 °C, 20 s at 50 °C and 30 s at 60 °C. To analyse the data, dynamic tube along with the slope correct was turned on and the cycle threshold was set at 0.01. Any sample having Cq score < 35 was considered positive, negative in case of no amplification or Cq score equal to zero and suspicious where Cq > 35.

**Table 1:** List of primers used for the *Wolbachia* screening in the BF

Strain	Primer and probe (5′–3′)	Reference
*w*AlbB	GF: GGTTTTGCTGGTCAAGTA	[39]
	BR: GCTGTAAAGAACGTTGATC	
	FAM_5′: TGTTAGTTATGATGTAACTCCAGAA-TAMRA	
*w*Mel	WD0513_F: CAAATTGCTCTTGTCCTGTGG	[26]
	WD0513_R: GGGTGTTAAGCAGAGTTACGG	
	WD0513_Probe_Cy5′: TGAAATGGAAAAATTGGCGAGGTGTAGG-BHQ	
*w*MelPop	IS5_F: CTCATCTTTACCCCGTACTAAAATTTC	[26]
	WD1310_R: TCTTCCTCATTAAGAACCTCTATCTTG	
	IS5_Probe_5′-Joe: TAGCCTTTTACTTGTTTCCGGACAACCT-TAMRA	

### *Wolbachia* tissue invasion after adult and pupal microinjection

BF were collected 9 days after adult injection and 13 days after pupal injection and rinsed with 80% ethanol followed by three sterile water washes. The head, thoracic muscle, midgut, fat body and ovaries were dissected aseptically from the flies under a stereomicroscope in a laminar flow cabinet and tested for *Wolbachia* by real-time PCR as described in the foregoing section.

### Fluorescence *in situ* hybridisation (FISH)

FISH was carried out to visualise *Wolbachia* distribution in female BF post-adult microinjection using a method slightly modified from that of Koga et al. [52]. Briefly, for the whole-mount assay, 10 BF infected with *w*Mel and *w*MelPop were collected six days post-injection and fixed in Carnoy’s solution (a mixture of chloroform, ethanol and acetic acid at a ratio of 6:3:1, respectively) overnight. Flies were washed the next day sequentially in 100%, 80% and 70% ethanol, and stored in 10% H_2_O_2_ in 100% ethanol for 30 days to quench the autofluorescence. Preserved flies were subsequently washed three times with 80% ethanol, 70% ethanol, and PBST× (0.8% NaCl, 0.02% KCl, 0.115% Na_2_HPO_4_, 0.02% KH_2_PO_4_, 0.3% Triton X-100) and pre-hybridised with hybridisation buffer (4× SSC, 0.2 g/ml dextran sulphate, 50% formamide, 250 μg/ml Poly A, 250 μg/ml salmon sperm DNA, 250 μg/ml tRNA, 100 mM DTT, 0.5× Denhardt’s solution) without probe two times for 15 min each. The insects were then incubated with hybridisation buffer and *Wolbachia 16S* rRNA probes overnight [53]. The next morning, samples were washed three times with PBSTx, three times for 15 min each and finally incubated in PBSTx containing DAPI (10 mg/ml) for 30 min. Samples were then rewashed with PBSTx, covered with ProLong Diamond Antifade Mountant (Thermo Fisher Scientific, Brisbane, Australia) and photographed using a confocal microscope.

### *Wolbachia* quantification

DNA was extracted from whole female BF post-adult and pupal injection using an Isolate II Genomic DNA extraction kit (Bioline). Six flies were assayed at 0, 3, 5, 7, 9 and 11 days post- adult injection and 0, 3, 7, 9, 13 and 15 days post-pupal microinjection to determine relative *Wolbachia* density. Real-time PCR assays were carried out in triplicate to amplify the *Wolbachia wsp* gene [54] and host reference gene *GAPDH* (378 F: 5′-CCG GTG GAG GCA GGA ATG ATGT-3′; 445 R: 5′-CCA CCC AAA AGA CCG TTG ACG-3′) on a Rotor-Gene Q Instrument (Qiagen). Reactions were run in a total volume of 10 μl having 5 μl Rotor-Gene SYBR® Green PCR Kit (Qiagen), 0.3 μl each of 10 µM forward and reverse primer and 2 µl of genomic DNA. Negative and positive PCR controls were included in all runs. Amplification was conducted for 5 min at 95 °C followed by 45 cycles of 10 s at 95 °C, 15 s at 55 °C and 15 s at 69 °C, acquiring on the green channel at the end of each step. Finally, *Wolbachia* density was calculated relative to host *GAPDH* using the delta-delta CT method [55]. Kruskal–Wallis and Dunn’s multiple comparison tests were carried out using GraphPad Prism 8 software to test for change in the *Wolbachia* density in injected flies.

### Survival assay

Two to three-hour-old female adult BF were injected with *Wolbachia* (*w*AlbB, *w*Mel and *w*MelPop) or SPG buffer (injected control) as described above and placed in triplicate cages containing ten flies each. Flies were cultured under laboratory conditions in small cages and mortality was noted every 12 h. Dead flies were later tested for *Wolbachia* infection individually using real-time PCR as described above. The survival assay for microinjected pupae was carried out as per the adult assay except that the number of flies in each cage was 20 (10 male and 10 female). Log-rank (Mantel–Cox) test was carried out using GraphPad Prism 8 software to test for difference in survival of BF post-*Wolbachia* infection.

### Adult emergence rate post-pupal microinjection with *Wolbachia*

Data from five independent pupae-microinjected batches were used to analyse the effect of *Wolbachia* on adult emergence. All three *Wolbachia* strains were injected in parallel to the buffer-injected controls. The number of injected pupae varied amongst batches between 77–205 for *w*Mel, 98–145 for *w*AlbB and 82–148 for *w*MelPop. The emergence of adults was recorded each day and the ratio of total emerged to number of injected pupae was calculated to determine the final percentage of emergence. The effect of *Wolbachia* on pupal emergence was analyzed using one-way ANOVA followed by multiple comparison Tukey’s test using GraphPad Prism 8 software.

### Total egg production post-pupal microinjection with *Wolbachia*

The effect of *Wolbachia* on the number of eggs produced by females after pupal microinjection was assessed in triplicate with ten females per cage. Buffer-injected females were used as controls and number of eggs laid and females surviving were counted every 24 h to estimate eggs laid per day per female. If any females died during the assay, the number of eggs was adjusted to account for this. Dead females were later tested for the presence of *Wolbachia* using real-time PCR. One-way ANOVA followed by multiple comparison Tukey’s test was carried out using GraphPad Prism 8 software to test for difference in egg production.

## Results

### Embryonic microinjection of buffalo flies

**Fig. 1:**
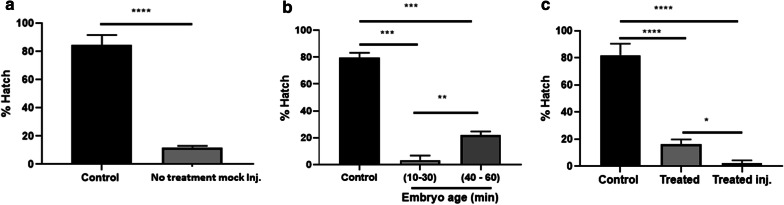
Challenges with buffalo fly embryonic microinjection. **a** Embryonic microinjection without *Wolbachia* had a detrimental effect on embryo hatching. **b** 40–60 min-old embryos survived injection better than 10–30 min-old embryos. **c** Eggs were dechorionated by treating with 2.5% sodium hypochlorite for 30 s and covered with 2:1 mix of halocarbon oil 700 and 27 to prevent desiccation. Eggs were sensitive to treatment and survival decreased further with the injection. Error bars are SEM. Analysis was by Fisher’s exact test in (**a**) and Tukey’s multiple comparison test in (**b**) and (**c**). ****P < 0.0001

Of a total of 2036 eggs microinjected with the *w*AlbB strain, only 10 developed through to adult flies (six females and four males) and no infection was detected in any of the adults. Microinjecting buffalo flies is particularly difficult because of the tough chorion surrounding the egg. We observed a significant detrimental effect of injection on embryo survival and hatching (Fisher’s exact test: *P* < 0.0001) (Fig. 1a) and identified that older eggs (40–60 min) had a better injection survival rate, 21.96% compared to 3.4% for younger eggs (10–30 min) (Tukey’s multiple comparison test: *P *= 0.010) (Fig. 1b). A number of other variations of the technique were tested to improve the survival rate of eggs post-microinjection. These included dechorionation of the eggs with 2.5% sodium hypochlorite for 30 s to soften the chorion, partial desiccation to reduce hydrostatic pressure in the eggs and increase space for the retention of larger volumes of injectate and the use of halocarbon oil (2:1 mix of halocarbon 700 and 27) to prevent desiccation of the eggs. None of these treatments markedly improved survival post-microinjection (2.33%) and they also appeared to reduce egg survival in uninjected eggs (16.33%) (one-way ANOVA: *F*_(2, 6)_= 181.6, *P* < 0.0001) (Fig. 1c).

### *Wolbachia* dynamics and tropism post-adult injection

**Fig. 2:**
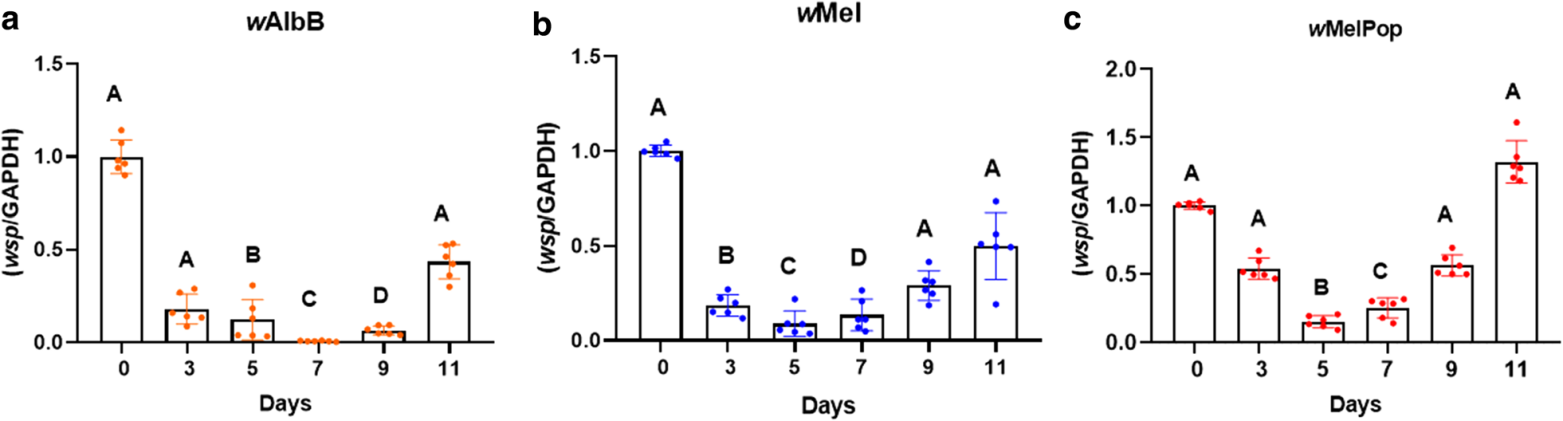
*Wolbachia* dynamics post-adult microinjection of female buffalo flies assessed using real-time PCR. *Wolbachia* dynamics measured over eleven days post-injection by analysing *n *= 6 for each day (**a–c**). Here, mean *Wolbachia* density is expressed relative to the host genome. Kruskal–Wallis test and Dunn’s multiple comparison test were used to compare titres at day 0. All error bars are SEM. Bars with different letters in each graph are significantly different

The growth kinetics of *Wolbachia* were studied in injected female flies by quantifying *Wolbachia* on days 3–11 compared to day 0 (day of injection). Overall, the pattern showed an initial significant decrease in *Wolbachia* density to approximately day 5 followed by subsequent growth and increase in bacterial titre to day 11 in all three strains (Kruskal–Wallis H-test: *χ*^2^= 31.18, *df *= 5, *P* < 0.0001) (Fig. 2a–c).

**Fig. 3:**
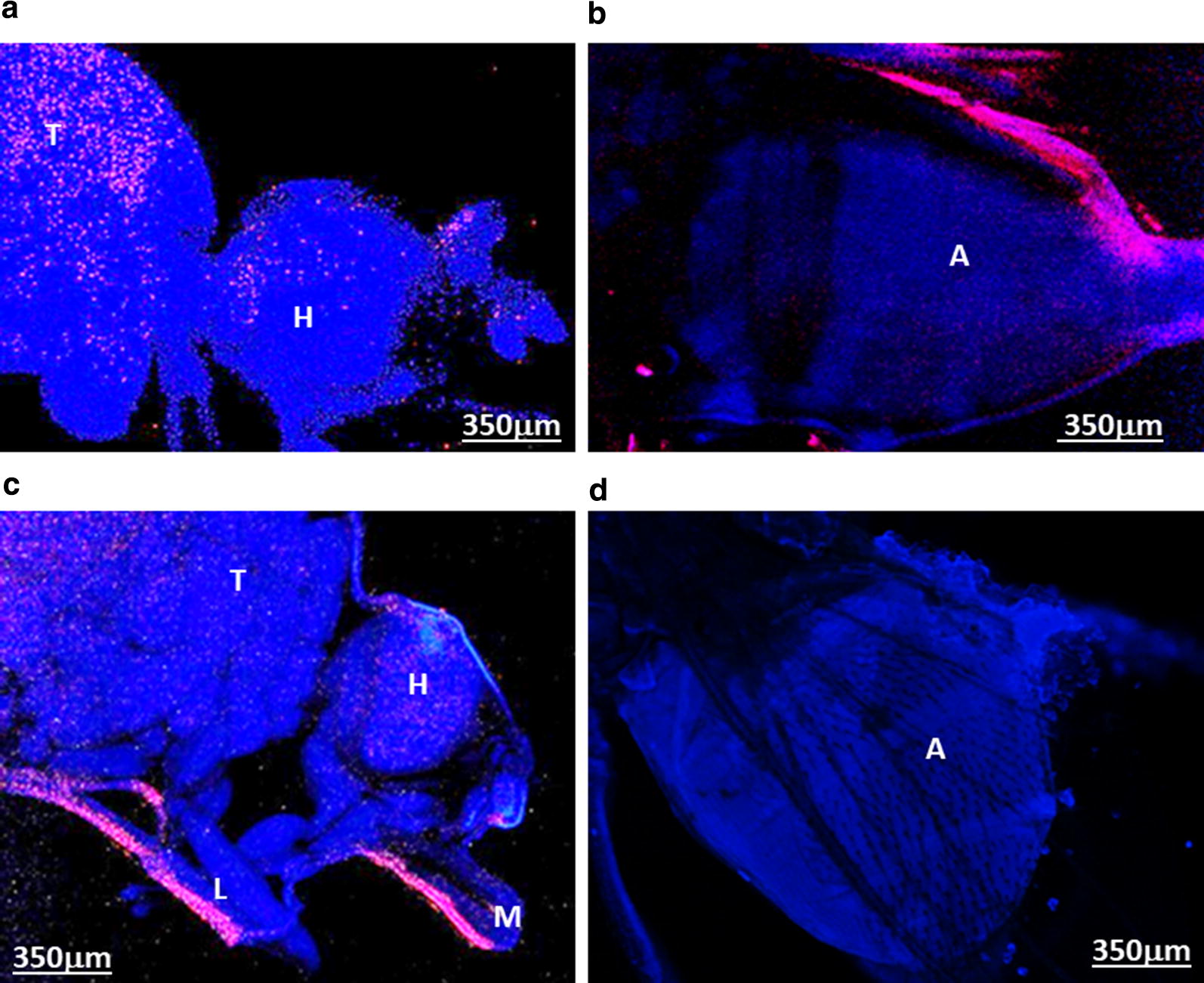
Fluorescence *in situ* hybridisation images showing localisation of *Wolbachia* six days post-adult injection. *Wolbachia* is distributed throughout the BF (blue: host, red: *Wolbachia*). **a***w*Mel in head and thorax. **b***w*MelPop in the abdominal region. **c***w*MelPop in the head, mouthparts, thorax and leg. **d** Control no probe. *Abbreviations*: T, thorax; H, head; A, abdomen; M, mouthparts; L, leg

**Fig. 4:**
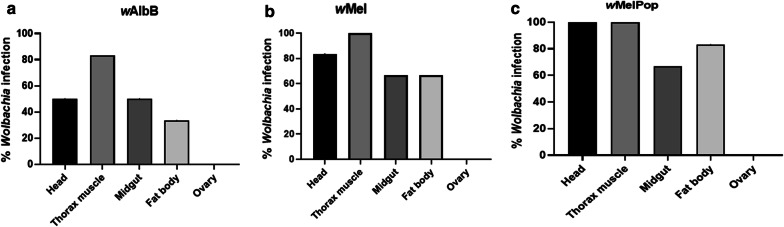
*Wolbachia* tropism post-adult microinjection of female buffalo flies assessed using real-time PCR. *Wolbachia* tropism in female (*n *= 6) nine days post-adult injection (**a–c**). None of the *Wolbachia* strains was found in the ovaries. Bars represent SEM

Significant variation in *Wolbachia* growth dynamics after injection required a better understanding of tissue tropism. Hence, fluorescence *in situ* hybridisation (FISH) was carried out on whole mounted BF and dissected ovaries to visualise the localisation of *w*Mel and *w*MelPop *Wolbachia* six days after injection (Fig. 3). No infection in the germline tissue was evident in any of the six samples analysed from each strain. However, *Wolbachia* was widely distributed in somatic tissues including the thoracic muscle, head, abdominal area, proboscis and legs (Fig. 3). The PCR results for *Wolbachia* growth in flies (Figs. 2, 3) suggest that the use of FISH at 6 days post-injection was too early to determine the final distribution of *Wolbachia*. Hence, we studied tissue invasion and the detailed distribution of *Wolbachia* in adult flies by real-time PCR after dissecting out the thoracic muscle, midgut, fat bodies, ovary and head at nine days post-adult injection (Fig. 4a–c). *Wolbachia* were found to be replicating in all somatic tissues with *w*AlbB having an infection percentage of 33–83% (*n *= 6) and *w*Mel and *w*MelPop between 66–100% (*n *= 6). No infection was found in germline tissues. However, on a few occasions first-generation flies from adult injection with *w*AlbB, *w*Mel,and *w*MelPop were found positive with infection percentages of 5%, 22% and 10%, respectively, suggesting transmission *via* the germline tissues in these instances (see Table 2).

**Table 2:** Summary of pupal and adult injections. G_0_ here represents the initially injected adults and adults emerged from injected pupae. G_1_ and G_2_ represents first generation and second generation respectively. Infection was determined using real-time strain-specific *Wolbachia* assays

Injection type	Strain	Total injected	G_0_ (infected/total tested) (% infection)	G_1_ (infected/total tested) (% infection)	G_2_ (infected/total tested) (% infection)	
Embryonic	*w*AlbB	2036 (12 batches)	Adult: 0/10 (0%)			
Adult	*w*AlbB	378 (19 batches)	Adult: 118/126 (95.93%)	Adult: 5/89 (5.6%)	Adult: not tested; Egg: 0/50 (0)	
Adult	*w*Mel	441 (17 batches)	Adult: 117/123 (95.12%)	Adult: 27/119 (22.68%)	Adult: 0/25 (0%); Egg: 0/100 (0%)	
Adult	*w*MelPop	417 (15 batches)	Adult: 103/106 (96.26%)	Adult: 10/91 (10.98 %)	Adult: 2/60 (3.3%)	
Pupal	*w*AlbB	676 (5 batches)	Adult: 82/90 (91.22%); Egg: 4/40 (10%)	Adult: 0/20 (0); Egg: 0/50 (0)		
Pupal	*w*Mel	820 (6 batches)	Adult: 82/82 (100%)	Adult: 2/9 (22); Egg: not tested		
Pupal	*w*MelPop	741 (5 batches)	Adult: 88/92 (95.65%); Egg: 0/30 (0)	Adult: 0/23 (0); Egg: not tested		

### Effect of *Wolbachia* on the survival of flies post-adult injection

**Fig. 5:**
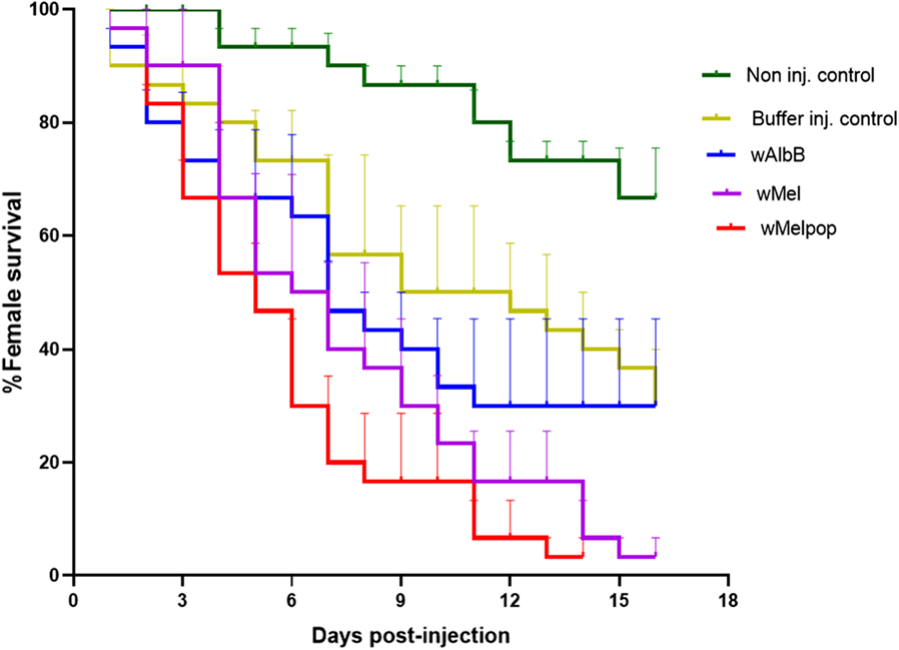
Survival of female buffalo flies post-adult injection with *Wolbachia*. Triplicate cages of adult flies each containing ten females were maintained under lab culturing conditions. The number of dead flies were recorded until all died. A significant reduction in survival was observed in *w*Mel (*P* < 0.0005) and *w*MelPop (*P* < 0.0001) injected flies by Log-rank (Mantel–Cox) tests

In order to understand the population dynamics of the flies inside the cage, survival assays were performed. The results revealed that by day seven less than 20% of the *w*MelPop and less than 50% of *w*Mel and *w*AlbB injected flies were alive (Fig. 5). Both *w*MelPop (log-rank statistic= 16.92, *P* < 0.0001) and *w*Mel (log-rank statistic= 11.96, *P *= 0.0005) significantly reduced longevity of female BF. However, there was no significant effect of the *w*AlbB strain in comparison to the control injected flies (log-rank statistic= 0.25, *P *= 0.62).

### *Wolbachia* dynamics and tropism post-pupal microinjection

**Fig. 6:**
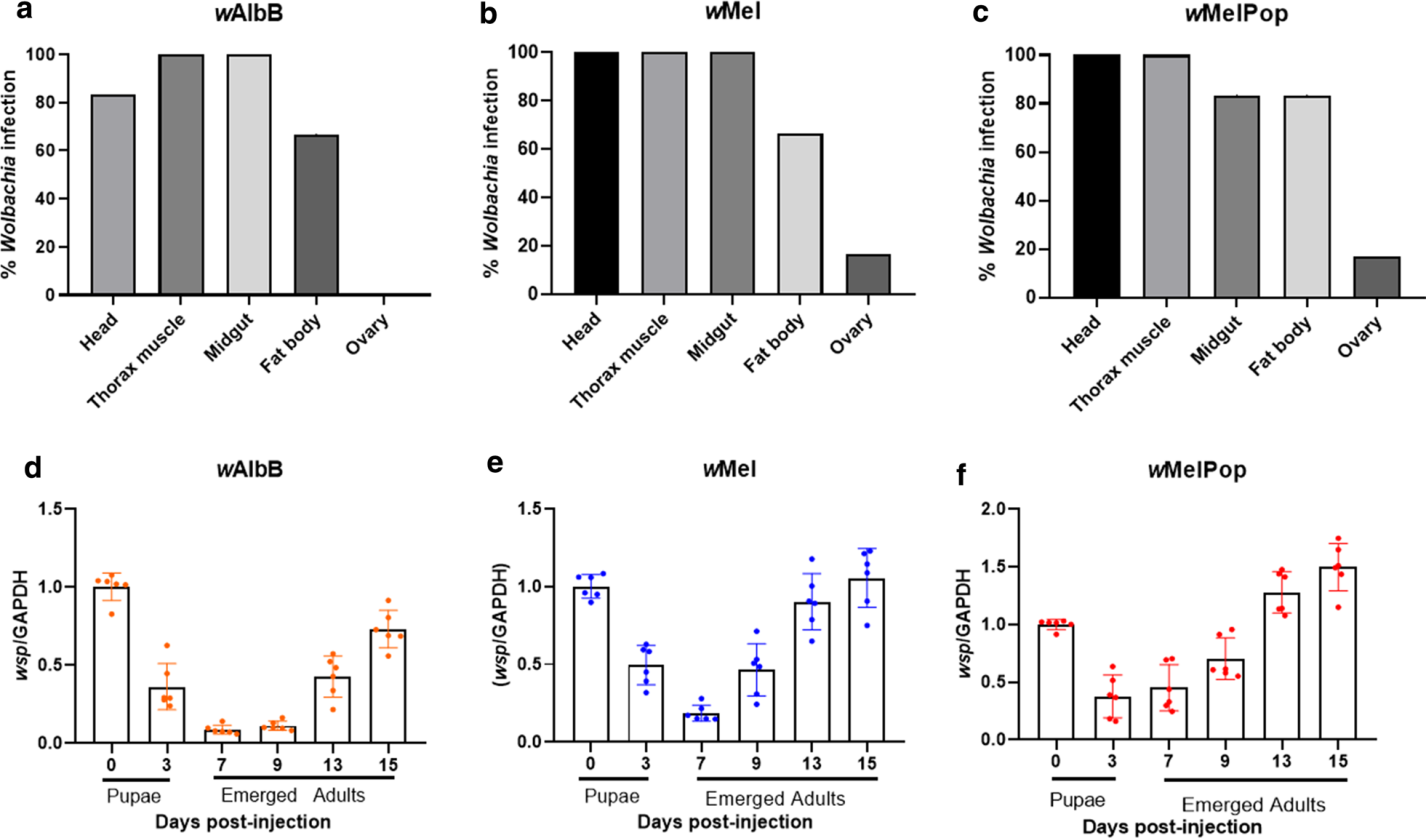
*Wolbachia* tropism and dynamics post-pupal microinjection of female buffalo flies assessed using real-time PCR. **a**–**c***Wolbachia* tropism in female BF (*n *= 6) 13 days post-pupal injection. Ovary infection was detected in *w*Mel and *w*MelPop injected flies. **d**–**f***Wolbachia* dynamics measured over 15 days post-injection. Here, mean *Wolbachia* density is expressed relative to the host genome. Kruskal–Wallis and Dunn’s multiple comparison tests were used to compare titres to those at day 0. Bars with different letters are significantly different (*P* < 0.05). Scale on the Y-axis for *w*MelPop (**f**) is different to that for the other two strains (**d, e**) indicating faster growth rate with *w*MelPop

A similar quantitative assay to that used for injected adult BF was carried out to track the dynamics and tropisms of the three *Wolbachia* strains post-pupal injection. The extra time in the pupal phase resulted in 66–100% infection in the somatic tissue with *w*AlbB and *w*Mel (*n *= 6) and 83–100% with *w*MelPop (*n *= 6) 13 days post-pupal injection (Fig. 6a–c). Furthermore, in 16% of cases the ovaries of females injected with *w*Mel and *w*MelPop *Wolbachia* were found to be infected. Also, two first-generation flies from *w*Mel-injected pupae and four eggs from *w*AlbB-injected pupae were found positive for *Wolbachia* infection (Table 2). Analysis of *Wolbachia* dynamics showed approximately the same pattern as for adult injection, where density initially decreased in the first seven days, then significantly recovered by day 9 in *w*Mel (Kruskal–Wallis H-test: *χ*^2^= 29.61, *df *= 5*, P* < 0.0001) and day 13 in *w*MelPop and *w*AlbB post-pupal injection (Kruskal–Wallis H-test: *χ*^2^= 32.12, *df *= 5*, P* < 0.0001) (Fig. 6d–f).

### Effect of *Wolbachia* on survival of buffalo flies post-pupal microinjection

**Fig. 7:**
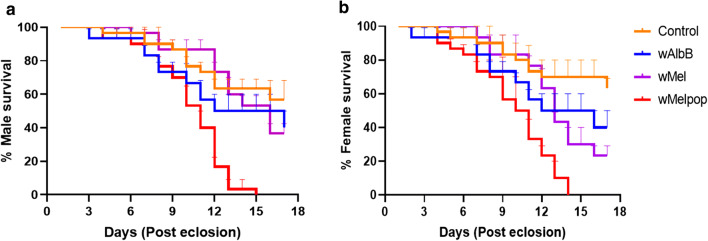
Survival of buffalo flies post-pupal injection with *Wolbachia*. Triplicate cages of flies eclosed from pupae on the same day (10 males and 10 females per cage) were maintained in laboratory culturing conditions. Mortality was recorded daily until all flies were dead. Log-rank (Mantel–Cox) showed a significant reduction in the male *w*MelPop (*P* < 0.0001) and female *w*MelPop (*P* < 0.0001) injected flies

A significant decrease in the longevity of BF post-pupal injection was found in both sexes of *w*MelPop-injected BF (male: log-rank statistic= 20.25, *P* < 0.0001, female: log-rank statistic= 29.04, *P* < 0.0001), but the effect was not significant with the two other strains [*w*AlbB: male (log-rank statistic= 2.267, *P *= 0.132), female (log-rank statistic= 3.275, *P *= 0.071); *w*Mel: male (log-rank statistic= 3.027, *P *= 0.1545), female (log-rank statistic= 3.467, *P *= 0.063)] (Fig. 7).

### Effect of *Wolbachia* on adult emergence rate

**Fig. 8:**
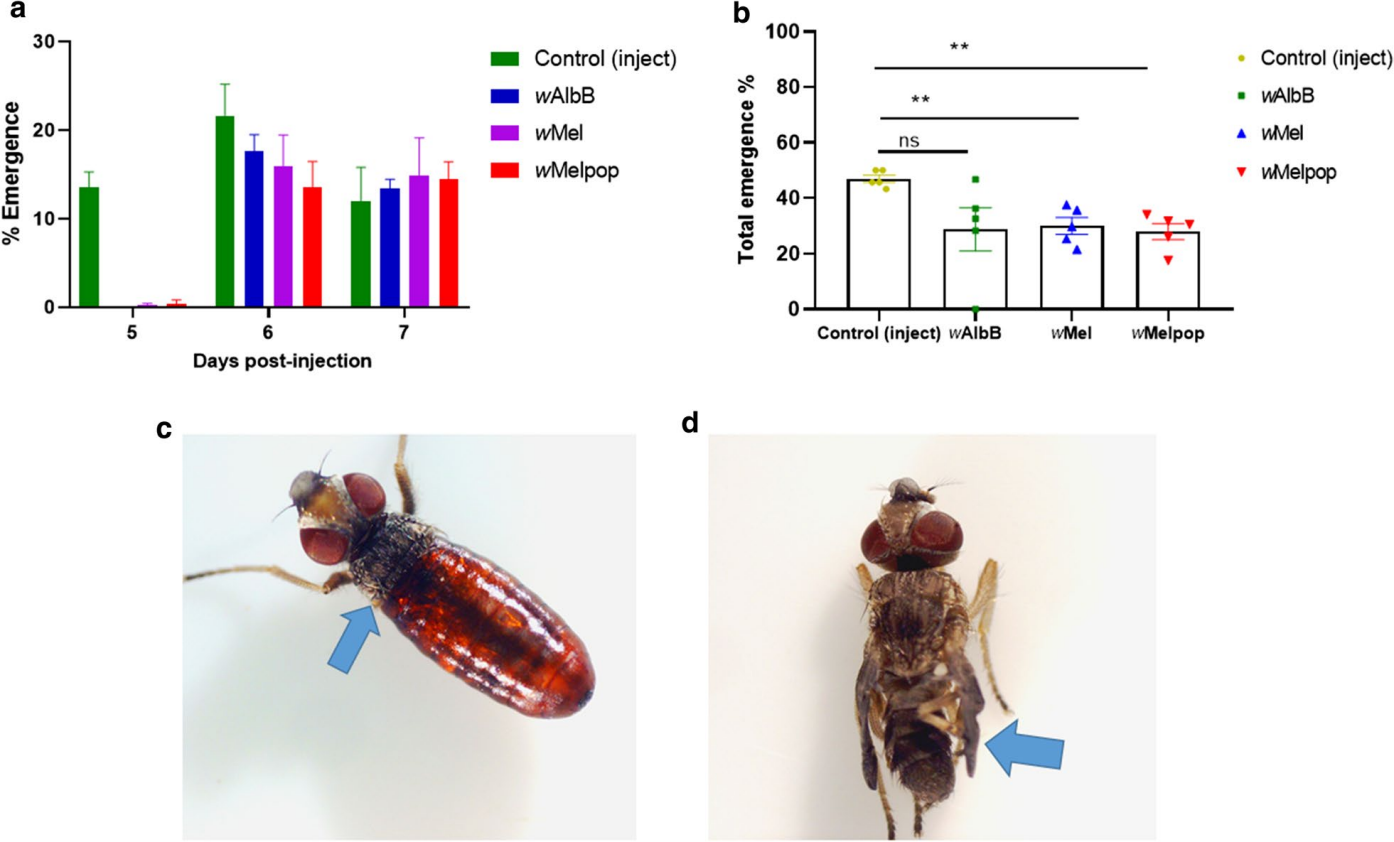
Fitness effects on buffalo fly post-pupal injection with *Wolbachia*. **a***Wolbachia* delayed adult emergence. **b** A significant decrease in adult emergence was observed in *w*Mel (*P *= 0.0030) and *w*MelPop (*P *= 0.0011) injected pupae when analysed using Tukey’s multiple comparison test. Nearly 5% of *w*MelPop flies either failed to completely eclose from the pupal case or had deformed wings

Infection of the somatic tissues by *Wolbachia* can have consequences on physiological processes. Non-injected control flies emerged from pupae after 3–7 days, whereas mock-injected control flies emerged from 5 to 7 days, *w*AlbB after 6–7 days and *w*Mel- and *w*MelPop-injected flies at 5–7 days post-injection (Fig. 8a). It is important to note that emergence in *w*Mel- and *w*MelPop-injected flies was less than 2% on day 5. Overall, there was significant decrease in the percent emergence of *w*Mel-injected (30.01 ± 3.91) (Tukey’s multiple comparison test, *P *= 0.0030) and *w*MelPop-injected flies (27.98 ± 3.92) (Tukey’s multiple comparison test, *P *= 0.0011) compared to the control injected flies (46.95 ± 4.15), but no significant difference was observed with the *w*AlbB-injected flies (Tukey’s multiple comparison test: *P *= 0.77) (Fig. 8b). Nearly 5% of the flies that emerged from the *w*MelPop-injected pupae were too weak to completely eclose from the pupal case and had deformed wings (Fig. 8c, d).

### Effect of *Wolbachia* on egg production

**Fig. 9:**
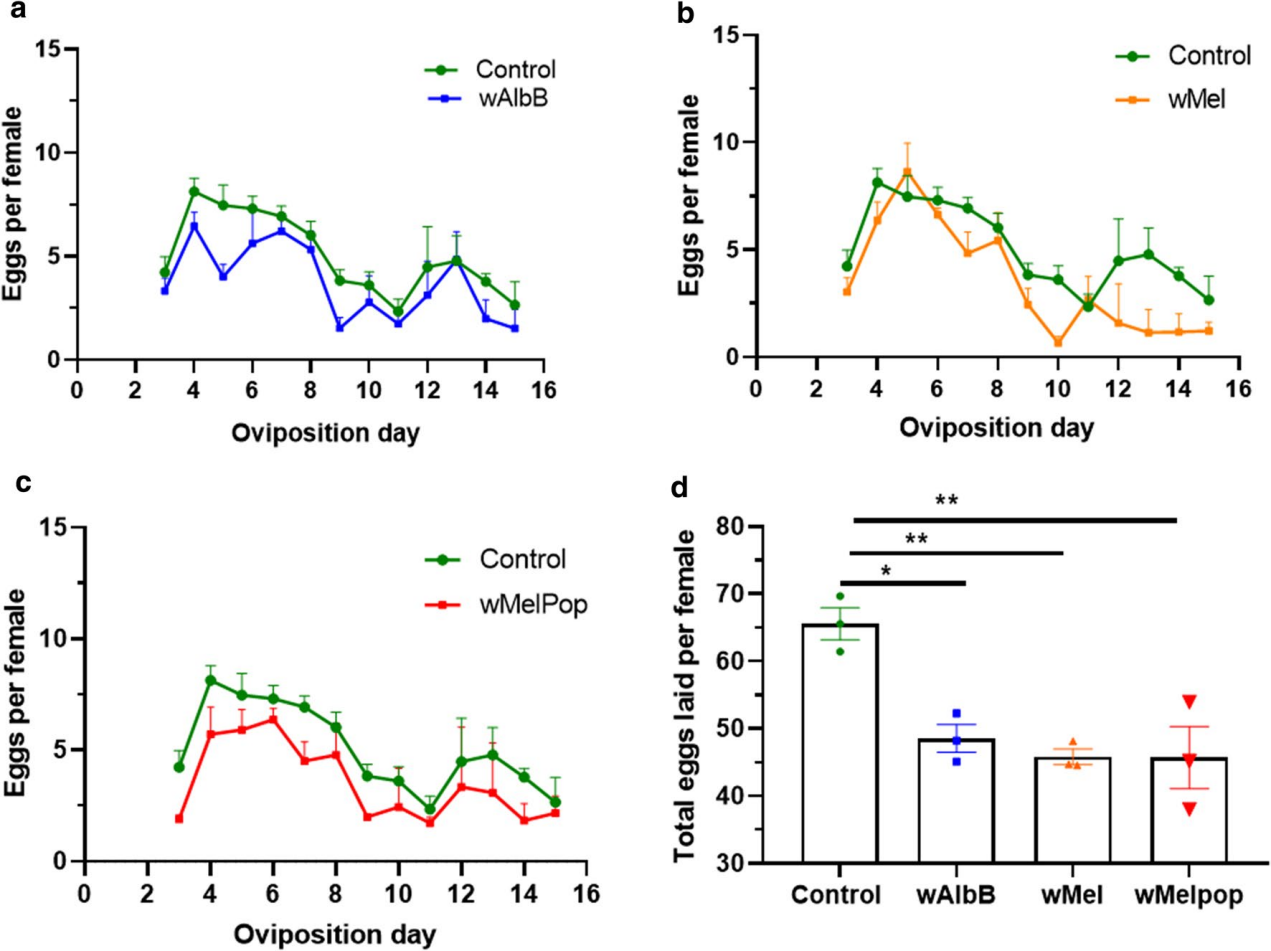
Fecundity of buffalo flies post-pupal injection with *Wolbachia*. Flies started laying eggs from day 3 post-emergence and continued until day 16. Eggs laid from triplicate cages each having ten females was recorded every day for (**a**) *w*AlbB (**b**) *w*Mel and (**c**) *w*MelPop. (**d)** A significant difference between the total number of eggs laid per female over 13 days was found in flies infected with *w*AlbB (*P *= 0.0123), *w*Mel (*P *= 0.0052) and *w*MelPop (*P *= 0.0051) (Tukey’s multiple comparison test)

Difference between infected females and non-infected females in egg production was also analysed following pupal injection with the three different strains of *Wolbachia*. Over 14 days there was a significant reduction in the total number of eggs laid by females infected with *w*AlbB (*P *= 0.012), *w*Mel (*P *= 0.0052) and *w*MelPop (*P *= 0.0051) in comparison with the mock-injected flies (Fig. 9).

## Discussion

Embryonic microinjection is by far the most frequently used technique to develop *Wolbachia* transinfected insect lines, mainly because *Wolbachia* injected into the germ cells of the developing embryo provides a direct route for infection of the germ tissues in the early stage of differentiation [36]. However, this technique is also the most challenging step because the invasive procedure of egg microinjection can result in high mortality of eggs and optimal methods differ for different insect species [36, 56, 57]. Another disadvantage of this technique is that inability to determine the sex of an embryo prior to injection means that approximately half of the injected flies will be males that do not transmit *Wolbachia* to the next-generation [36]. This means that many thousands of eggs must often be microinjected using specialised equipment before successful *Wolbachia* transinfection is achieved [36] and as male embryos cannot be identified, half of this effort is functionally wasted. With BF, less than 1% of more than 2000 embryos we injected subsequently hatched because the tough chorion of BF eggs caused difficulties with needle penetration, rapid blunting and high breakage rate of microinjector needles, frequent chorion tearing, and embryo damage. Treatment with sodium hypochlorite to soften the chorion, prior partial desiccation of eggs to reduce hydrostatic pressure, and the use of halocarbon oils to prevent egg desiccation during injection did not markedly improve the survival rate. Similar difficulties were experienced when attempting to use microinjection for gene transfection in closely related *H. i. irritans* eggs. In this instance, the researchers opted to use electroporation, which is unsuitable for the introduction of bacteria [58].

Although embryonic microinjection has been the primary method used to develop transinfected insects, adult microinjection can be advantageous in that females can be selected for injection [36]. Further, adult microinjection can be performed using a simple syringe and small-bore needles delivering higher volumes of *Wolbachia* to overcome the host immunological response [36]. Our results with adult injection of *Wolbachia* were promising. Despite that injections in first few batches were made mainly with *Wolbachia* grown in *D. melanogaster* cells (*w*AlbB, *w*Mel and *w*MelPop strain), not previously adapted in *Haematobia* cells, infection rates and persistence in the injected flies were high (generally > 90%). In a few batches, transmission to the next-generation was confirmed.

As oviposition by BF may begin as early as three days after eclosion from the pupae and continue until death, knowledge of *Wolbachia* distribution and dynamics in injected females was critical for us to identify the optimal timing for collecting infected eggs for the establishment of an infected colony (11–15 days). *Wolbachia* density significantly decreased to day five possibly due to host immune response [48] but recovered by day eleven after injection. A similar result was obtained when *w*MelPop and *w*AlbB were injected into *Anopheles gambiae* adult mosquitoes [59]. The initial host immune response was anticipated as the densities of *w*AlbB, *w*Mel and *w*MelPop *Wolbachia* in *Haematobia* cells were also observed to initially decrease, possibly due to an innate immune response mediated by the Imd pathway [48]. Real-time PCR analysis of dissected tissues nine days after injection showed *Wolbachia* to be present in all the vital somatic tissues (head, thoracic muscle, midgut and fat body), except for the ovarial tissues, suggesting that *Wolbachia* might need extra time to infect the ovaries. However, injection with *w*AlbB, *w*Mel and *w*MelPop *Wolbachia* caused > 40% death in flies by day seven post-injection, further reducing the likelihood of collecting infected eggs. Therefore, we hypothesised that microinjecting 1–2 h-old pupae would give more time than with adult microinjection for *Wolbachia* to multiply, spread and establish in the ovaries. Pupal injection has previously been conducted with *Trichogramma* wasps and resulted in successful ovarian infections and persistence of *Wolbachia* in the wasp colony for 26 generations [60].

With BF*, w*Mel and *w*MelPop overcame host immune responses and established in both somatic and germline tissues. Further, in two instances, next-generation (G1) BF from *w*AlbB and *w*Mel injected pupae were positive for *Wolbachia*, indicating next-generation transmission as a result of pupal injection. The main disadvantages of pupal injection in comparison with adult injection were limitation on the volume of *Wolbachia* that could be injected and inability to distinguish female from male pupae for injection.

The *w*MelPop strain is a virulent type of *Wolbachia*, and its over replication in somatic tissues and brain cells, known in other infected insects [61, 62], may have been the reason for the early death of BF. Further, in the studies of *Wolbachia* kinetics we found a higher density of *w*MelPop than with the other two strains following both adult and pupal injection. Reduction in the longevity of infected *Ae. aegypti* mosquitoes caused by infection with *w*MelPop, decreasing the potential extrinsic incubation time for the dengue virus, was one of the characteristics that led to the hypothesis that *w*MelPop infection would reduce dengue spread [63]. Infection with *w*MelPop could also markedly reduce BF life span and their ability to transmit *Stephanofilaria* sp. nematodes. These nematodes have been implicated in the development of buffalo fly lesions, a significant production and welfare issue in north-Australian cattle [64]. *Stephanofilaria* has an extrinsic incubation period of up to 3 weeks in *Haematobia* spp. [65] and the life-shortening effects of *Wolbachia* shown in our study could markedly reduce the vector competency of infected flies. There is also the possibility the *Wolbachia* infection could more directly compromise the vector competency of BF for *Stephanofilaria* sp., as has been seen in the case another filarial nematode, *Brugia pahangi* transmitted by mosquitoes and in the case transmission of the dengue virus by *Ae. aegypti* [66, 67].

Fecundity of insects has a significant influence on population dynamics of insect populations [68] and when *Wolbachia*-infected males are released CI, vertical transmission, and a relatively higher fertile egg production by infected females in comparison with uninfected flies may increase the chances of successful establishment of *Wolbachia* in a new host population [69]. *Wolbachia* have been found to enhance and reduce egg production depending upon both the strain of the *Wolbachia* and the host [21, 69–74]. We found that *w*AlbB, *w*Mel and *w*MelPop significantly reduced total egg production in pupal injected flies. Also, *Wolbachia* infection caused delayed and decreased adult emergence of BF post-pupal injection. *Wolbachia* being an endosymbiont lacks nutritional biosynthetic pathways and depends on its host for wide range of nutrition [75]. Hence, the fitness costs observed in injected BF could be the result of competition between high density of *Wolbachia* and BF for nutritional resources such as amino acids and lipids [75, 76]. Another possibility could be that as *Wolbachia* was found in all of the critical tissues involved in the endocrine cascades for egg production and maturation in insects (midgut, neuron, fat bodies and ovary), it interfered with egg production by this means [77]. In addition, delayed larval development associated with *w*MelPop infection has been documented in mosquitoes on a number of occasions [23, 25]. If these deleterious effects are a consistent feature of *Wolbachia* infection in BF, they could have a significant impact in altering population dynamics or even crashing BF populations [23, 78]. For instance, female BF lay eggs in fresh cattle manure pats, where eggs take approximately seven days to develop into pupae depending upon the temperature and moisture content of the pat [79]. Prolonged larval development and time to eclosion of *Wolbachia*-infected BF, together with adult life span reduction might decrease overwintering and survival of BF, particularly during periods of unfavourable fly conditions and at the edge of the BF range.

## Conclusions

BF are competent hosts for the growth of *w*Mel, *w*MelPop and *w*AlbB *Wolbachia* strains and infection can induce a number of fitness effects in injected flies. However, embryonic injection proved challenging with BF and it was not possible to establish an infected isofemale line using this technique. Pupal and adult microinjection gave much higher fly survival rates, high titres of *Wolbachia* in somatic tissues and ovarian infection and transmission to the next-generation in a number of instances. Despite relatively limited testing, this gives hope for the establishment of sustainably *Wolbachia*-infected strains of BF and the future development of a *Wolbachia*-based area-wide control programme.

## Data Availability

Data supporting the conclusions of this article are included within the article.

## Abbreviations

BF: buffalo fly
FISH: fluorescent in situ hybridization
*GAPDH*: glyceraldehyde-3-phosphate dehydrogenase
*wsp*: *Wolbachia* surface protein
